# Long noncoding RNA SNHG14 facilitates colorectal cancer metastasis through targeting EZH2-regulated EPHA7

**DOI:** 10.1038/s41419-019-1707-x

**Published:** 2019-07-04

**Authors:** Wu Di, Xue Weinan, Li Xin, Yu Zhiwei, Gu Xinyue, Tong Jinxue, Li Mingqi

**Affiliations:** 1 Department of Colorectal Surgery, Harbin Medical University Cancer Hospital, Harbin, China; 2 Department of Cardiology, The Fourth Affiliated Hospital of Harbin Medical University, Harbin, China; 3 Digestive System Diseases Center, The Seventh Affiliated Hospital of Sun Yat-sen University, Guangzhou Shi, China

**Keywords:** Proteomics, Cancer genomics

## Abstract

Accumulating evidence suggested the participation of long noncoding RNAs (lncRNAs) in regulating various biological processes so as to affecting cancer progression. However, the functional role of most lncRNAs in colorectal carcer (CRC) is still largely covered. In the present study, we disclosed SNHG14 as a carcinogene in CRC development, as it was low-expressed in normal colon tissues but markedly upregulated in CRC cell lines. Besides, SNHG14 contributed to CRC cell proliferation, motility and EMT in vitro, and inhibition of it confined CRC tumor growth and liver metastasis in vivo. Next, the mechanistic investigations confirmed that SNHG14-promoted CRC progression was mediated by EPHA7, which was negatively regulated by SNHG14 in CRC via an EZH2-dependent way. Importantly, EZH2 was proved as a transcription factor of EPHA7 and functioned as a repressor in EPHA7 transcription by enhancing methylation on EPHA7 promoter. Meanwhile, SNHG14 increased EZH2 expression in CRC via stabilizing its mRNA by interacting with FUS, and via freeing its mRNA from miR-186-5p-induced silence. All in all, our observations demonstrated that SNHG14 serves as a facilitator in CRC through targeting EZH2-repressed EPHA7 by enhancing EZH2 via recruiting FUS and absorbing miR-186-5p, indicating a promising new road for CRC diagnosis and treatment.

## Introduction

Colorectal cancer (CRC) is the third most prevalent neoplasm worldwide, with more than one million people diagnosed as CRC annually^1^. Considering the high incidence and mortality, CRC is thought as a great threat to public health^2^. Recently, the CRC-associated morbidity and mortality in China have been revealed to be significantly risen^3^. Although increasing explorations into the molecules involved in CRC, as well as improvements in the diagnostics and therapeutic strategies^4,5^, the prognosis of CRC patients in China is still disappointing with nearly 191,000 cases of CRC-caused deaths each year^6^. Therefore, intensive researches are badly imperative to find out novel targets for the diagnosis and treatment of CRC.

Long noncoding RNAs (lncRNAs) are classified as a kind of noncoding RNAs (ncRNAs) with base pairs greater than 200^7^. Although lncRNAs were considered as transcriptional “noise” that had no biological functions^8^, amounting studies have uncovered the emerging role of lncRNAs in multiple cellular processes, such as cell differentiation, proliferation, migration, invasion and so on^9,10^. Based on these functions, lncRNAs have been revealed as key modulators in a large number of human diseases^11^, including various cancers^12–16^. Also, recent reports have unveiled several lncRNAs as oncogenic or tumor-suppressive genes in CRC^17^. For instance, SNHG5 contributes to CRC cell survival by targeting STAU1^18^. LINC01133 plays an antitumor part in CRC by interacting with SRSF6^19^. SNHG14 is a lately recognized lncRNA that serves as a contributor in several cancers^20–24^ and as a tumor suppressor in glioma^25^. Importantly, the probable implication of SNHG14 in CRC progression has been indicated by a recent report^26^. Therefore, we wondered the potential role of SNHG14 in CRC progression.

In this study, we aimed to figure out the specific function of SNHG14 in the development and progression of CRC, and the in-depth mechanism whereby SNHG14 affected CRC progression was explored as well.

## Materials and methods

### Cell culture

The normal human colorectal cell line NCM460 and five CRC cell lines (LoVo, SW620, SW480, HCT116 and HT-29) were purchased from the Cell Bank of Chinese Academy of Science (Shanghai, China). All the cells were grown in DMEM (Gibco, USA) with 10% fetal bovine serum (FBS; Sigma-Aldrich, St. Louis, MO, USA) and maintained at 37 °C in an atmosphere containing 5% CO_2_.

### Plasmid construction and cell transfection

The pcDNA3.1 vector (Invitrogen, USA) containing the full-length cDNA sequences of SNHG14, EPHA7, or EZH2 was applied to overexpress SNHG14, EPHA7, or EZH2, respectively. And short hairpin RNAs (shRNAs) targeting SNHG14 or EZH2 (shSNHG14 or shEZH2) were provided by GenePharma, while small interfering RNAs (siRNAs) against EPHA7 or FUS (si-EPHA7 or si-FUS) were constructed by Thermo Fisher Scientific. The empty pcDNA3.1 vector and scramble shRNA or siRNA were utilized as negative controls. Subsequently, LoVo and HT-29 cells were transfected with above plasmids as needed using Lipofectamine™ 2000 (Invitrogen, USA) following the manufacturer’s guides. After 48 h of transfection, cells were collected for further use.

### Quantitative real-time PCR (qRT-PCR)

Total RNA was isolated from cultured cells by the use of TRIzol reagent (Thermo Fisher Scientific, USA), and then reversely transcribed with the GoScript Reverse Transcription System (Qiagen GmbH, Germany). Afterwards, the qRT-PCR analysis was conducted on the ABI 7900 Detection System (Applied Biosystems, USA) by using the SYBR-Green PCR Master Mix kit (Takara, Dalian, China). Relative expression of genes normalized to GAPDH was calculated using 2^−ΔΔCt^ method.

### Colony formation assay

Eight hundred of cells were seeded into each well of the 12-well plates in triplicate, and then cultured for two weeks with the medium replaced every 3 days. Thereafter, the colonies containing more than 50 cells were fixed by methanol, stained using crystal violet (Sigma, USA) and counted manually. And the colony formation rate was calculated using formula as below: colony formation rate = (number of colonies/number of seeded cells) × 100%.

### CCK-8 assay

Cell Counting Kit 8 (CCK-8, Donjindo) was utilized to examine cell viability according to the manufacturer’s protocols. In brief, cells were placed into 96-well plates, with the initial concentration of 1 × 10^3^ cells/well. After being incubated for 24, 48, 72, and 96 h, cells in each well were added with CCK-8 solution and further incubated for 2 h. The absorbance at 450 nm was measured using a microplate reader (MRX; Dynex Technologies, West Sussex, UK).

### Transwell assay

Cell migration and invasion were assessed by conducting Transwell assays by the use of transwell chambers (BD Biosciences, San Jose, CA, USA). For cell migration assay, the upper chambers were added with cell suspension containing 1 × 105 cells, while the lower chambers were supplemented with 600 μl of DMEM containing 10% FBS. After incubation for 24 h, cells were fixated using methanol and stained by crystal violet. Finally, cells from five random fields were counted under a microscope. With respect to cell invasion assay, the upper chambers were precoated with Matrigel for 1 h at 37 °C, with the other procedures were consistent with the transwell migration assay.

### Chromatin immunoprecipitation (ChIP)

The ChIP assays were carried out using the Magna ChIP Kit (Millipore, Billerica, MA, USA) in the light of manufacturer’s instructions. Firstly, the crosslink between DNA and proteins was fixated by using formaldehyde for 30 min. Then the DNAs isolated from CRC cells were fragmented into 200–1000 bp using sonication. Subsequently, the fragmented DNAs were incubated overnight with protein A/G beads containing antibodies against EZH2 or IgG (negative control). The subsided DNA fragments were determined through qRT-PCR.

### Luciferase reporter assay

To examine the effect of EZH2 on EPHA7 transcription, as well as that of SNHG14 on EZH2 transcription, pGL3 plasmids containing firefly reporter were used to construct recombinant plasmids with EPHA7 or EZH2 promoter. Then the recombinant plasmids were transfected into appropriate HEK-293T cells (with altered EZH2 or SNHG14 expression) by using Lipofectamine™ 2000 (Invitrogen, USA). In order to detect the binding of miR-186-5p to SNHG14 or EZH2, the psiCHECK2 vector (Promega, Madison, WI) was applied to obtain SNHG14-WT, SNHG14-Mut, EZH2-WT, and EZH2-Mut, and then HEK-293T cells were cotransfected with above recombinant plasmids and miR-NC, miR-186-5p mimics, or miR-186-5p mimics together with pcDNA3.1/SNHG14. Forty-eight hours post transfection, the luciferase activities were evaluated with dual-luciferase reporter assay system (Promega, Madison WI, USA).

### RNA pull down assay

RNA pull down assays were performed as previously described^27^. In short, the biotinylated RNAs (Bio-NC, Bio-miR-186-5p-WT and Bio-miR-186-5p-Mut) incubated with cell lysates, followed by the incubation with streptavidin beads (Invitrogen). Next, the RNAs in complexes captured by streptavidin beads were washed, purified and detected by qRT-PCR.

### RNA immunoprecipitation (RIP)

The RIP assays were conducted by the use of a Millipore EZ-Magna RIP RNA-Binding Protein Immunoprecipitation kit (Millipore, Bedford, MA, USA) in line with the manufacturer’s recommendations. The precipitated RNAs were tested by qRT-PCR. Antibodies applied in RIP assays were as follow: anti-FUS (Abcam, ab23439), anti-Ago2 (Abcam, ab186733) and anti-IgG (Millipore, PP64). IgG was used as negative control and input as positive control. Experiments were conducted for three times.

### Western blot analysis

Western blot analysis was performed to evaluate the expression of genes at protein level. Total protein was extracted from cells or murine tumor tissues by using RIPA (Radio Immunoprecipitation Assay) lysis buffer with protease inhibitors, followed by the concentration determined with bicinchoninic acid (BCA; Beyotime, China) method. Thereafter, the separation of proteins was realized by 10% SDS-PAGE, with the separated proteins transferred to nitrocellulose (NC) membranes (Amersham Bioscience, UK) subsequently. Following being blocked by 5% skim milk, the membranes were incubated at 4 °C overnight with primary antibodies specifically against E-cadherin, N-cadherin, Vimentin, EZH2 and GAPDH (All from Abcam, Cambridge, UK). At length, the visualization of chemiluminescent signals was performed using ECL (Enhanced Chemiluminescent) detection reagents (Amersham Biosciences, Sweden). GAPDH acted as the internal control. Proteins in each sample were detected in triplicate.

### In vivo mice assays

The in vivo xenograft and metastasis assays were conducted according to the previous protocols^28^. Total of 24 BALB/C nude mice (6–8 weeks old; Laboratory Animal Center, Zhengzhou) were employed in the study. The LoVo cells transfected with shCtrl or shSNHG14 were inoculated into the left flank of mice or injected from the tail vein of mice to establish in vivo tumor growth or metastasis models, respectively. All experiments involved in the animal studies were carried out under the approval of the Institutional Animal Care and Use Committee (IACUC) at Harbin Medical University Cancer Hospital.

### Statistical analysis

Data collected from at least three independent experiments was all analyzed using SPSS 17.0 (SPSS Inc, USA), with the results represented as the mean ± SD Then differences were estimated by performing Student’s *t* test (between two groups) or one-way ANOVA (among at least three groups). Differences with a *P* value lower than 0.05 was defined as statistically significant.

## Results

### SNHG14 is highly expressed and facilitates malignant phenotypes in CRC cells

**Fig. 1:**
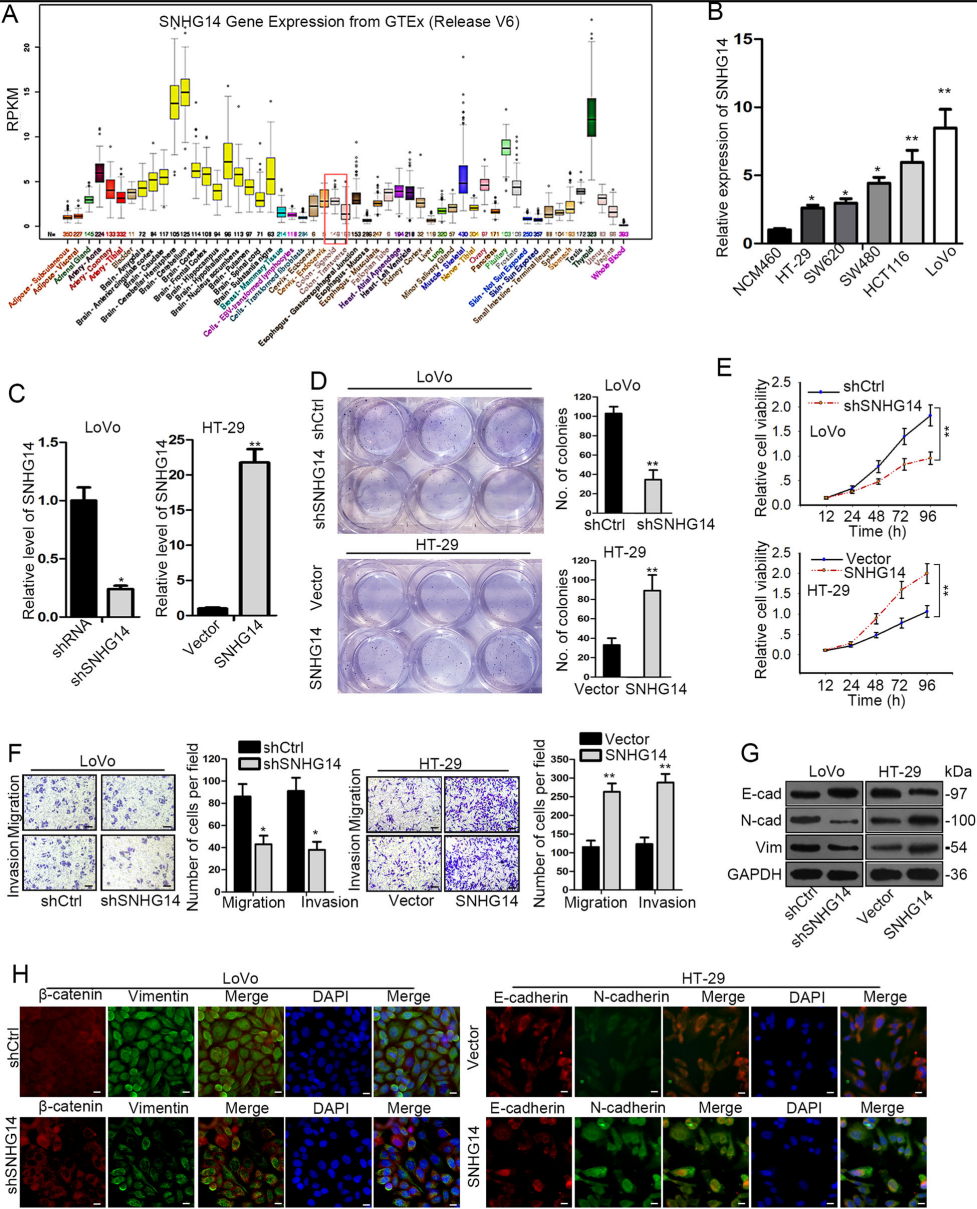
SNHG14 was highly expressed in CRC and promoted malignant phenotypes in CRC cells. **a** The expression profile of SNHG14 in 53 kinds of normal tissues from 570 donors was obtained from UCSC. **b** The level of SNHG14 in five CRC cell lines and the normal NCM460 cells was examined by qRT-PCR. **c** The transfection efficiency in LoVo and HT-29 cells after transfections was confirmed by qRT-PCR. **d**, **e** The proliferation of indicated CRC cells was evaluated by colony formation assay and CCK-8 assay. **f** The effect of SNHG14 on CRC cell migration and invasion was tested by transwell assay. **g**, **h** The expression of EMT-related proteins was detected through conducting western blot and IF (Immunofluorescence) analysis. ^*^P < 0.05, ^**^P < 0.01

To understand the role of SNHG14 in CRC, we first wondered whether SNHG14 was dysregulated in CRC. The UCSC (University of California, Santa Cruz) database revealed the apparent low expression of SNHG14 in normal colon tissues from 570 healthy donors (Fig. 1a). In contrast, compared with the normal human colonic cell line NCM460, we observed that the level of SNHG14 was distinctly enhanced in all the five CRC cell lines including HT-29, SW620, SW480, HCT-116, and LoVo, among which LoVo cells exhibited the highest endogenous SNHG14 expression while HT-29 cells showed the lowest conversely (Fig. 1b). Based on this, we suspected that SNHG14 might act as a carcinogene in CRC development.

In order to further confirm the function of SNHG14 in CRC progression, the loss- and gain-of-function assays were performed respectively after the expression of SNHG14 was silenced in LoVo cells or enhanced in HT-29 cells (Fig. 1c). As indicated in Fig. 1d, e, both the proliferation and viability of cells were controlled by SNHG14 inhibition but strengthened under SNHG14 overexpression. Similarly, knockdown of SNHG14 led to a suppressive effect on cell migration and invasion in LoVo cells, whereas ectopic expression of SNHG14 resulted in an opposite impact on the motility of HT-29 cells (Fig. 1f). Moreover, the expression of E-cadherin was elevated while that of N-cadherin and Vimentin reduced in response to SNHG14 knockdown, however, the protein levels of above three EMT-related genes were affected by SNHG14 upregulation in an absolute inverse way (Fig. 1g). Furthermore, the expression of E-cadherin and N-cadherin detected by IF staining was represented in consistent with above results (Fig. 1h). Therefore, we suggested that SNHG14 contributes to CRC cell proliferation, migration, invasion, and EMT.

### Depletion of SNHG14 restrains tumor growth and metastasis in vivo

**Fig. 2:**
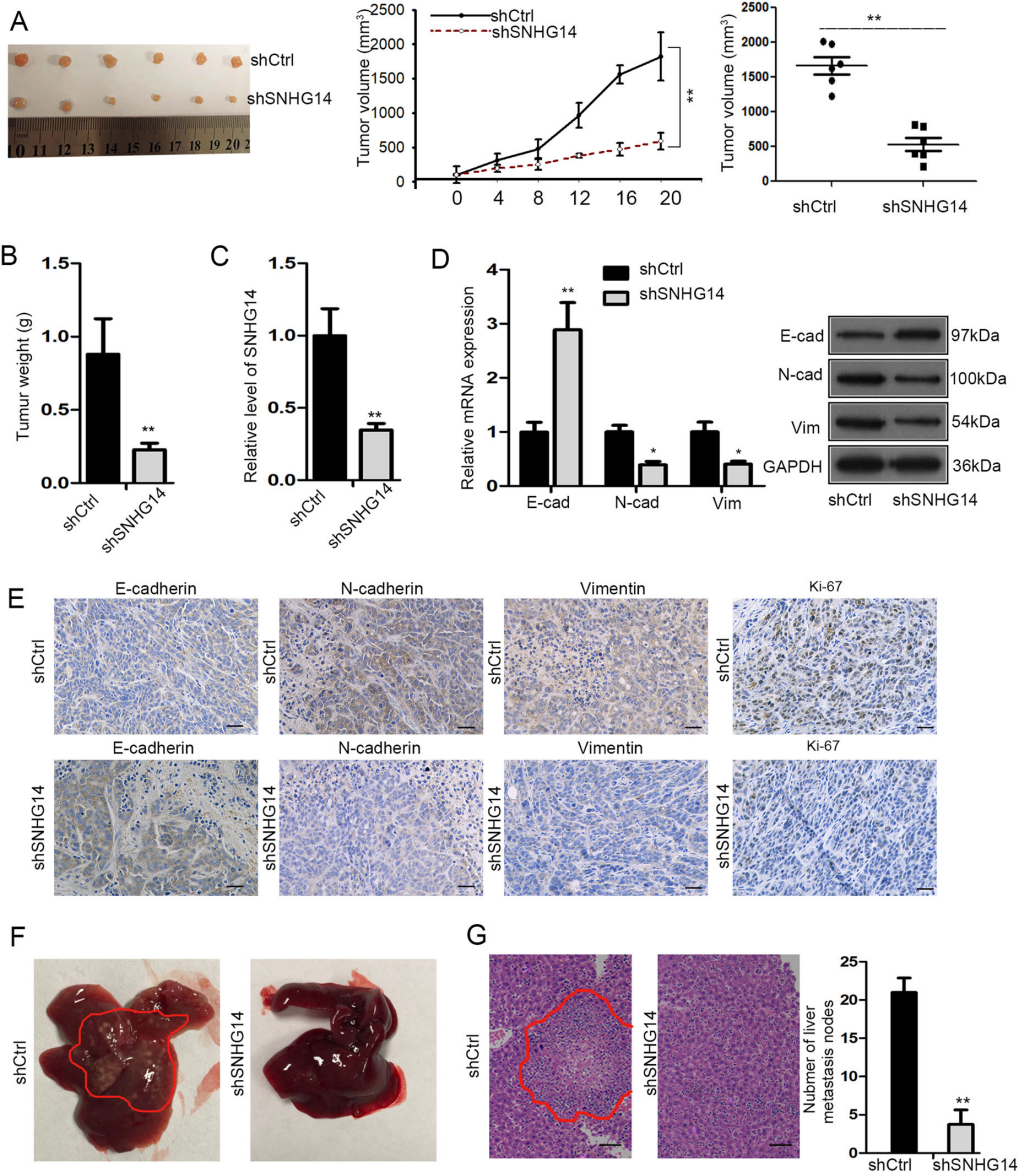
Silencing SNHG14 repressed CRC cell growth and metastasis in vivo. **a** Representative images and the volume of tumors derived from LoVo cells with or without SNHG14 inhibition. **b** Tumor weight of above two groups. **c** The expression of SNHG14 in tumors originated from shCtrl or shSNHG14-transfected LoVo cells was estimated by qRT-PCR. **d** qRT-PCR results of the expression of E-cadherin, N-cadherin and Vimentin in tumors in mice inoculated with shCtrl or shSNHG14-transfected LoVo cells. **e** The protein levels of E-cadherin, N-cadherin, Vimentin, and Ki67 in tumors from indicated cells were estimated by IHC. **f**, **g** The representative images (**f**) and HE staining of metastatic nodules (**g**) in livers of mice injected with shCtrl or shSNHG14-transfected LoVo cells. ^*^P < 0.05, ^**^P < 0.01

Subsequently, the in vivo experiments were conducted to further verify the oncogenic role of SNHG14 in CRC. Unsurprisingly, the tumors originated from SNHG14-silenced LoVo cells were observably smaller (with a significantly slowed growth rate and reduced size) and lighter (with lessened mean weight) than those from shCtrl-transfected cells (Fig. 2a, b). Meanwhile, the expression level of SNHG14 was validated as low-expressed in SNHG14 depletion-derived tumors (Fig. 2c). Meanwhile, the tumors from SNHG14-silenced group also represented increased expression of E-cadherin and decreased expression of N-cadherin and Vimentin in comparison with those from control group (Fig. 2d, e). Furtherly, the in vivo metastatic experiments demonstrated that the secondary tumors traveled to liver were much less in mice inoculated with SNHG14-depleted LoVo cells than those in mice with shCtrl-transfected LoVo cells (Fig. 2f, g). To be concluded, SNHG14 promotes tumor growth and metastasis in CRC.

### EPHA7 is involved in SNHG14-facilitated CRC progression

**Fig. 3:**
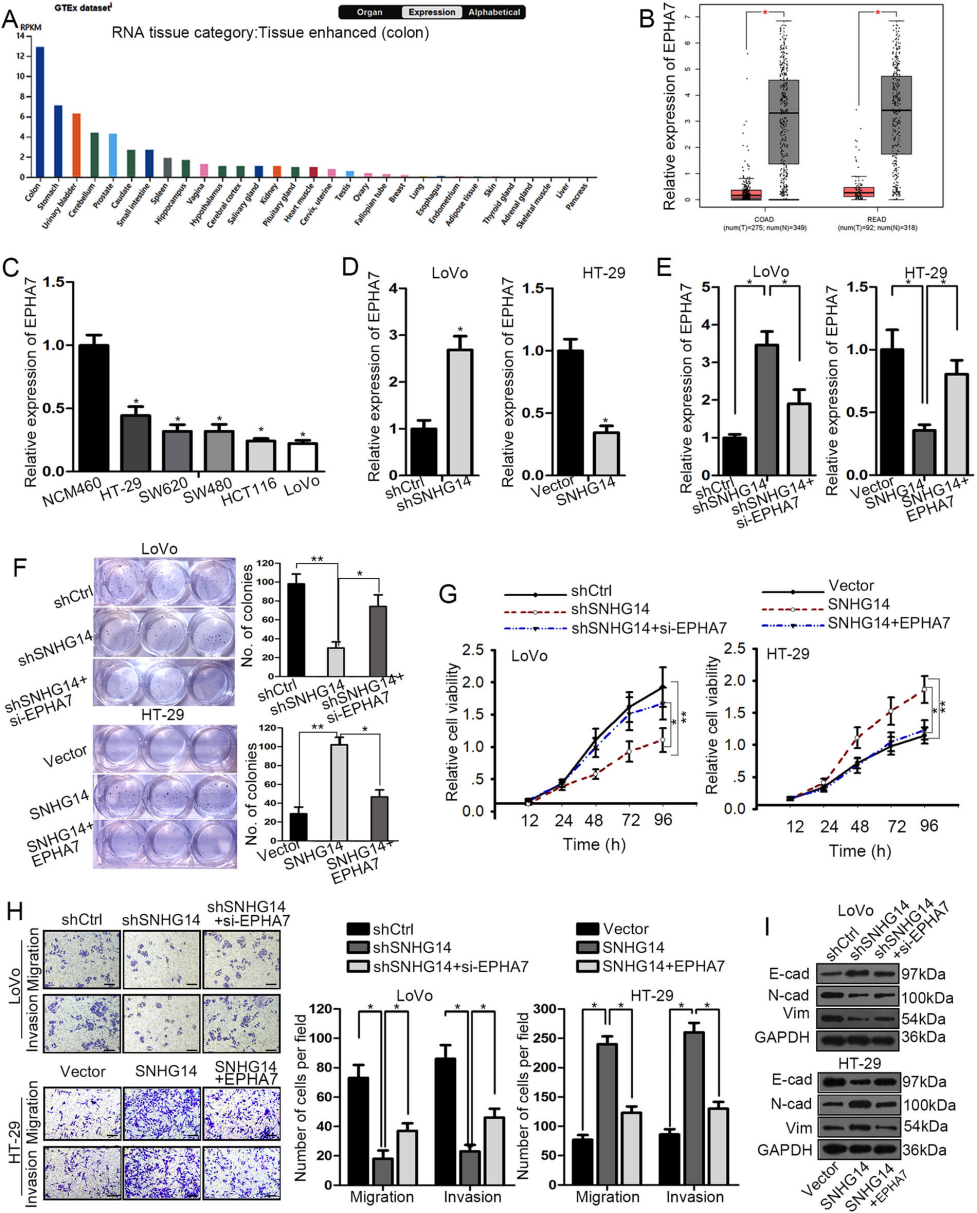
EPHA7 was implicated in SNHG14-regulated CRC development. **a** The expression of EPHA7 at protein level in normal tissues was suggested by online THPA. **b** TCGA database indicated the downregulation of EPHA7 in COAD and READ tissues compared to the normal tissues. **c** The qRT-PCR result of the expression level of EPHA7 in CRC cell lines and NCM460 cells. **d**, **e** The relative expression of EPHA7 in CRC cells under different transfections was assessed using qRT-PCR. **f**–**i** The impact of EPHA7 inhibition or overexpression on SNHG14 depletion- or upregulation-affected cellular processes in CRC cells was estimated by performing colony formation assay, CCK-8 assay, transwell assay and western blot. ^*^P < 0.05, ^**^P < 0.01

Recently, the important role of Eph family in cancer has been increasingly uncovered^29^. Here, we found that EPHA7, a member of ephrin receptor subfamily, was revealed as greatly highly expressed at protein level in normal colon tissues by The Human Protein Altas (THPA; website: https://www.proteinatlas.org/), with the data shown in Fig. 3a. Meanwhile, the TCGA database suggested that EPHA7 was downregulated in colon adenocarcinoma (COAD) tissues and rectum adenocarcinoma (READ) tissues, compared to the corresponding normal tissues (Fig. 3b). Also, we discovered a remarkable downregulation of EPHA7 in CRC cell lines relative to the normal NCM460 cells (Fig. 3c). Importantly, the expression level of EPHA7 was pronouncedly enhanced by SNHG14 silence but sharply diminished under SNHG14 overexpression (Fig. 3d), implying a negative regulation of SNHG14 on EPHA7 in CRC.

Next, we were curious about whether EPHA7 was implicated in the regulatory mechanism underlying SNHG14 affected CRC progression. Firstly, we confirmed that knockdown of EPHA7 offset the level of EPHA7 enhanced by SNHG14 inhibition, whereas ectopic expression of EPHA7 countervailed the SNHG14 upregulation-decreased EPHA7 level (Fig. 3e). Moreover, both the inhibited colony formation ability and viability in SNHG14-depleted LoVo cells were revived in response to the cotransfection of si-EPHA7, while the strengthened cell proliferation in SNHG14-overexpressed HT-29 cells were counteracted in face of enforced expression of EPHA7 (Fig. 3f, g). Furthermore, we demonstrated that the effect of SNHG14 suppression or upregulation on cell motility and EMT was also neutralized by EPHA7 coinhibition or co-overexpression, respectively (Fig. 3h, i). Thus, we drew a conclusion that SNHG14 aggravates CRC progression through an EPHA7-dependent way.

### EPHA7 is epigenetically silenced by EZH2

**Fig. 4:**
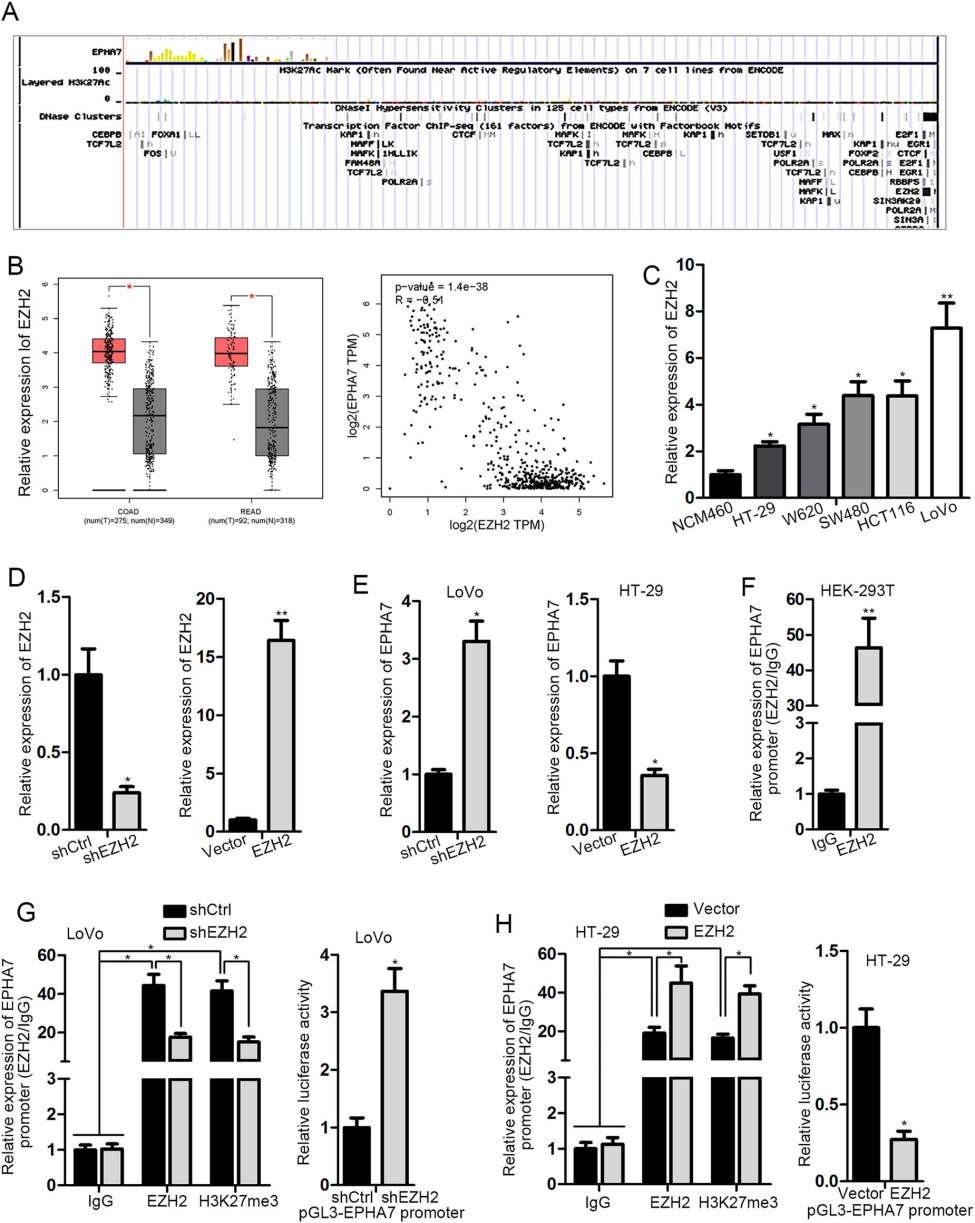
EPHA7 was transcriptionally silenced by EZH2 through elevating the methylation on EPHA7 promoter. **a** UCSC indicated that EZH2 was one of the transcription factors of EPHA7. **b** The expression of EZH2 in COAD and READ tissues and the correlation between the expression of EPHA7 and EZH2 in COAD were predicted by TCGA database. **c** Relative expression of EZH2 in CRC cell lines and NCM460 cells detected by qRT-PCR. **d**, **e** The expression of EZH2 and EPHA7 in EZH2-inhibited LoVo cells or EZH2-overexpressed HT-29 cells was assayed with qRT-PCR. **f** The binding of EZH2 to EPHA7 promoter was validated via conducting ChIP assay. **g**, **h** The influence of EZH2 on EPHA7 transcription was evaluated by luciferase reporter assay and ChIP assay. ^*^P < 0.05, ^**^P < 0.01

Furtherly, we aimed to explore the upstream molecule of EPHA7. Given that EPHA7 downregulation in CRC is revealed to be due to the hypermethylation in its promoter^30^, we suspected that EPHA7 might be transcriptionally regulated by EZH2, as indicated by UCSC database (http://genome.ucsc.edu/) (Fig. 4a). Meanwhile, EZH2 was suggested to be upregulated in COAD and READ, and a negative correlation between EPHA7 expression and EZH2 in COAD was also implied by GEPIA (Fig. 4b). Besides, we proved a significant upregulation of EZH2 in all the five CRC cell lines in comparison with the NCM460 cells (Fig. 4c). In addition, an exact negative regulation of EZH2 on EPHA7 expression was confirmed in CRC cells, as the level of EPHA7 was greatly boosted in EZH2-inhibited LoVo cells but distinctly reduced in EZH2-upregulated HT-29 cells (Fig. 4d, e). Moreover, we verified that EPHA7 promoter was obviously concentrated in EZH2-precipitated complex (Fig. 4f). Furthermore, depletion of EZH2 markedly mitigated the EZH2-enriched EPHA7 promoter, and also led to a hypomethylation on EPHA7 promoter and an enhancement on the luciferase activity of EPHA7 promoter (Fig. 4g). Conversely, overexpression of EZH2 resulted in lessened EPHA7 promoter immuno-precipitated by EZH2, hypermethylation on EPHA7 promoter and reduced luciferase activity of EPHA7 promoter (Fig. 4h). Altogether, these results explained that EZH2 negatively modulates EPHA7 transcription by promoting methylation on EPHA7 promoter.

### SNHG14 stabilizes EZH2 mRNA through recruiting FUS

**Fig. 5:**
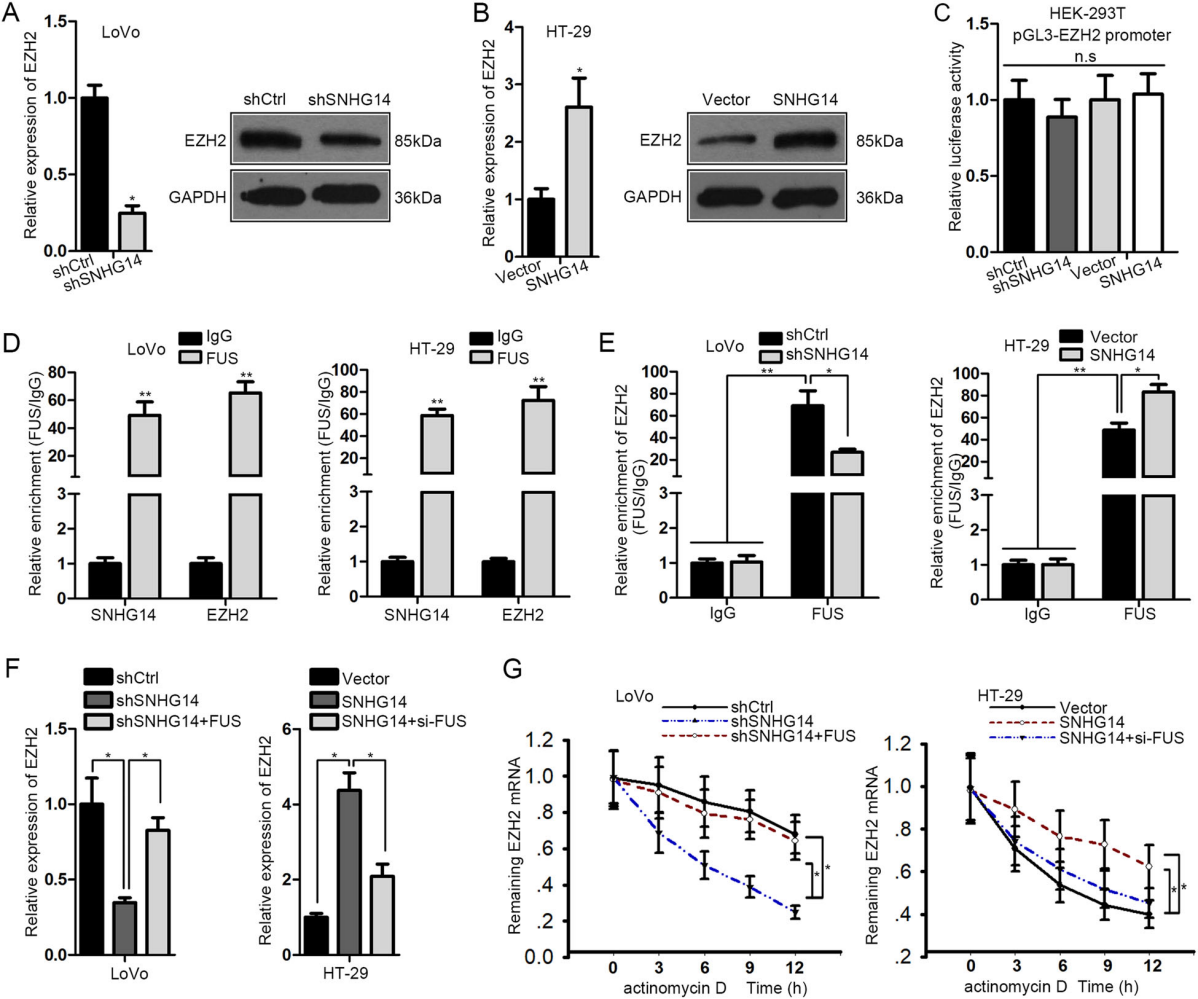
SNHG14 stabilized EZH2 mRNA by interacting with FUS. **a**, **b** The effect of SNHG14 on EZH2 expression in indicated CRC cells was detected by qRT-PCR and western blot analysis. **c** Luciferase reporter assay was conducted to assess the influence of SNHG14 on EZH2 transcription. **d** RIP assay validated the common interaction of FUS with both SNHG14 and EZH2 mRNA. **e** The impact of SNHG14 on FUS-interacted EZH2 mRNA was estimated by preforming RIP assay. **f** qRT-PCR result of EZH2 level in indicated CRC cells. **g** The degradation rate of EZH2 mRNA in indicated CRC cells under the treatment of actinomycin D was tested by qRT-PCR. ^*^P < 0.05, ^**^P < 0.01

Next, we investigated the in-depth mechanism whereby SNHG14 affected EZH2/EPHA7 axis in CRC. Firstly, we validated that the expression of EZH2 (at both the mRNA and protein levels) was decreased in the context of SNHG14 repression but increased in face of SNHG14 overexpression (Fig. 5a, b), proving the positive regulation of SNHG14 on EZH2 in CRC. In addition, the luciferase activity of EZH2 promoter was nearly unchanged either by SNHG14 downregulation or upregulation (Fig. 5c). Therefore, we suspected that SNHG14-regulated EZH2 expression in CRC at a posttranscriptional level.

Recently, emerging evidence has proved the participation of an RNA-binding protein (RBP) in lncRNA-regulated gene expressions^31^. The starBase 2.0 (http://starbase.sysu.edu.cn/starbase2/index.php) predicted that FUS was the shared RBP that interacted with both SNHG14 and EZH2 mRNA. Therefore, we wondered whether FUS was implicated in SNHG14-enhanced EZH2 expression. First of all, the RIP assays confirmed that both SNHG14 and EZH2 were dramatically harvested by FUS in LoVo and HT-29 cells (Fig. 5d). Also, the level of FUS-bond EZH2 mRNA was noticeably reduced upon SNHG14 silence but enhanced under SNHG14 overexpression (Fig. 5e). Additionally, the influence of SNHG14 inhibition or stimulation on EZH2 expression in CRC cells could be partially offset in the context of FUS upregulation or downregulation correspondingly (Fig. 5f). Intriguingly, the degradation rate of EZH2 mRNA accelerated by SNHG14 silence was normalized by forced expression of FUS, whereas SNHG14 upregulation retarded the degradation of EZH2 mRNA was rescued by FUS knockdown (Fig. 5g). Collectively, SNHG14 modulates the stabilization of EZH2 mRNA through a FUS-mediated manner.

### SNHG14 upregulates EZH2 by releasing miR-186-5p-targeted EZH2 mRNA

**Fig. 6:**
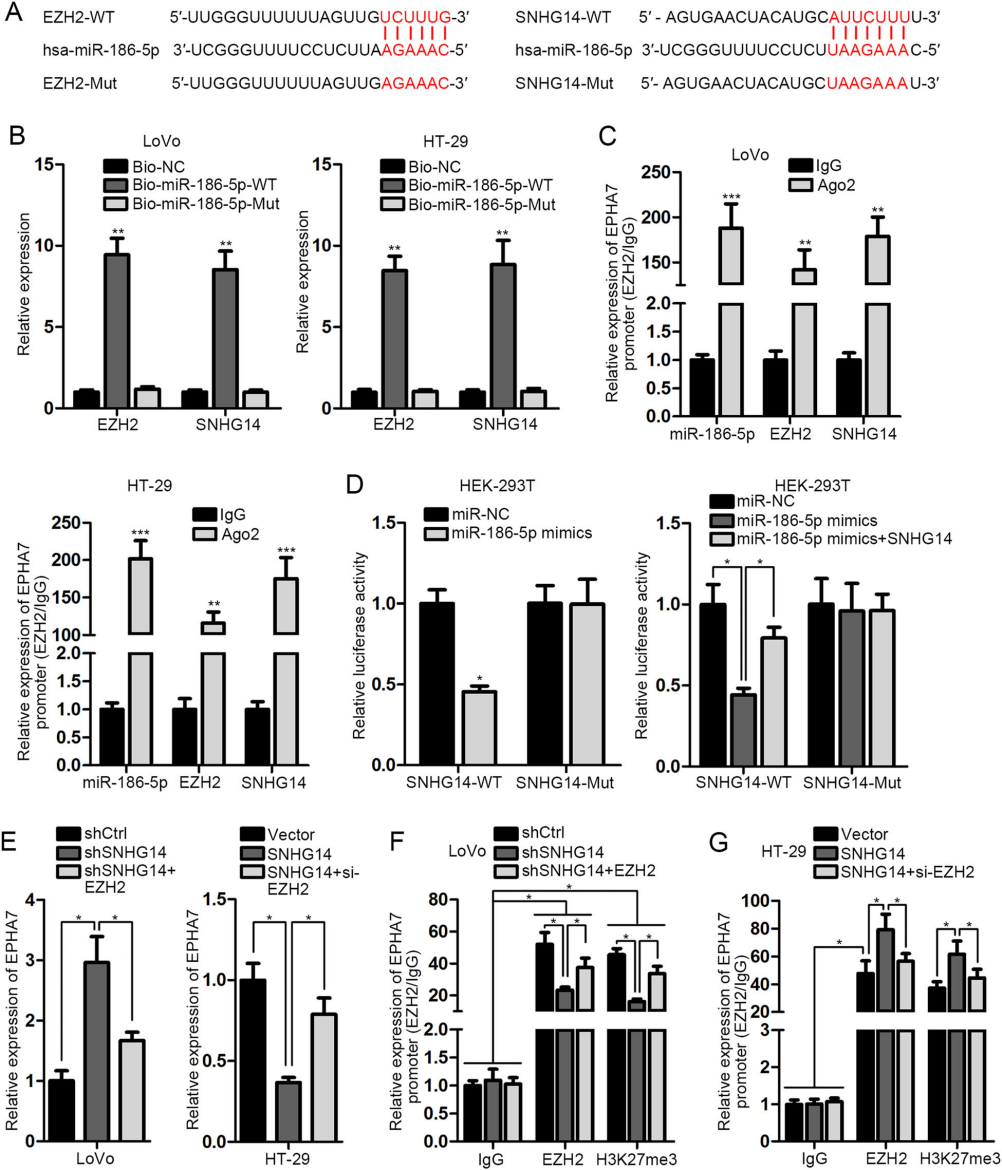
SNHG14 was a ceRNA of EZH2 by absorbing miR-186-5p. **a** The sequences of wild-type and mutant SNHG14 or EZH2 as well as miR-186-5p predicted by starBase 2.0. **b**, **c** The interaction of SNHG14, miR-186-5 p and EZH2 mRNA was proved by RNA pull down and RIP assays. **d** Luciferase reporter assays explained that SNHG14 and EZH2 mRNA bond with miR-186-5p by a competitive manner. **e**–**g** The impact of SNHG14 on EPHA7 expression was verified by carrying out qRT-PCR and ChIP. ^*^P < 0.05, ^**^P < 0.01, ^***^P < 0.001

At the same time, the online starBase predicted that both SNHG14 and EZH2 were the target of miR-186-5p, a previously-identified tumor suppressor in CRC^32^, with the corresponding sequences exhibited in Fig. 6a. Next, we demonstrated that both SNHG14 and EZH2 were pulled down by Bio-miR-186-5p-WT, rather than Bio-NC or Bio-miR-186-5p-Mut (Fig. 6b). Besides, the enrichment of SNHG14, miR-186-5p, and EZH2 mRNA was all easily observed in anti-Ago2 induced immunoprecipitations (Fig. 6c), suggesting the co-existence of above three molecules in RNA-induced silencing complex. Moreover, the results of luciferase reporter assay explained the specific inhibition of miR-186-5p upregulation on the luciferase activity of SNHG14-WT and EZH2-WT, whereas the miR-186-5p upregulation-reduced luciferase activity of EZH2-WT could be recovered in response to SNHG14 overexpression (Fig. 6d), proving the competitive relationship between SNHG14 and EZH2 mRNA when binding with miR-186-5p. More importantly, the negative regulation of SNHG14 on EPHA7 was verified to be mediated by EZH2, since EZH2 overexpression or silence countervailed the promotion or suppression of SNHG14 inhibition or upregulation on EPHA7 (Fig. 6e). Moreover, we certified that SNHG14 impaired EPHA7 expression through regulating EZH2-modulated methylation on EPHA7 promoter (Fig. 6f, g). All in all, our findings unveiled that SNHG14 facilitates CRC progression through targeting EZH2-repressed EPHA7.

## Discussion

In the past decades, accumulating reports have recommended lncRNAs as crucial regulators in gene expression and cancer development^33–36^. In this basis, lncRNAs associated cancer initiation and progression need to be identified to provide new targets for cancer therapy^37^. SNHG14 is a newly discovered lncRNA whose tumorigenic role has already been illustrated in gastric cancer^23^, clear cell renal cell carcinoma^22^, bladder cancer^21^, cervical cancer,^20^ and non-small cell lung cancer^24^, and its anti-tumor role in glioma has also been revealed previously^25^. In the present study, it is the first to recognize SNHG14 as a oncogene in CRC cell growth and metastasis by both the in vitro and in vivo experiments, suggesting the high potential of SNHG14 as an effective biomarker for the diagnosis and treatment of CRC patients.

The EPH-receptor subfamily of the protein-tyrosine kinases family and the ephrin ligands play important roles in cell–cell interactions by initiating a unique bidirectional signal transduction cascade between cells expressing EPHs and ephrins^38^. Recently, the implication of EPHs and ephrins in tumor development and metastasis has been largely suggested in various human cancers^39^, including CRC^40,41^. The Eph-receptor (EPHA7) is proved as a tumor suppressor in follicular lymphoma^42^, but as a carcinogene in human laryngeal squamous cell carcinoma^43^. Also, the upregulation of EPHA7 in most of the human lung tissues was confirmed by Tsuboi et al.^44^. Currently, EPHA7 was validated to be involved in SNHG14-regulated CRC progression, and the negative regulation of SNHG14 on EPHA7 was disclosed to be mediated by EZH2 via modulating the methylation of EPHA7 promoter, consistent with a previous finding that EPHA7 downregulation in CRC is due to its hypermethylation^30^.

Increasingly, studies elucidate that lncRNAs can regulate gene expression through various mechanisms^45,46^. Herein, we first proved that SNHG14 regulated EZH2 in CRC at a posttranscriptional level, on account of no influence on EZH2 transcription validated by luciferase reporter assays. On the one hand, RBPs are also identified as the important regulators in gene expression^47^, and the involvement of RBPs in lncRNAs-regulated gene expression has been largely unmasked recently^48,49^. In this study, we found the interaction of both SNHG14 and EZH2 with FUS, an oncogenesis-associated RBP that participates in transcriptional regulation and RNA processing^50,51^. In addition, the involvement of FUS in lncRNA-affected gene expression has been revealed previously^27,52^, in consistent with the findings in this study that SNHG14 strengthened the stabilization of EZH2 mRNA through a FUS-mediated manner. On the other hand, the ceRNA network has been largely proposed as one of the main mechanism by which lncRNAs exert the function in cancer^53,54^. Currently, we further explored that SNHG14 was involved in a ceRNA regulatory network by competitively binding miR-186-5p with EZH2.

**Fig. 7:**
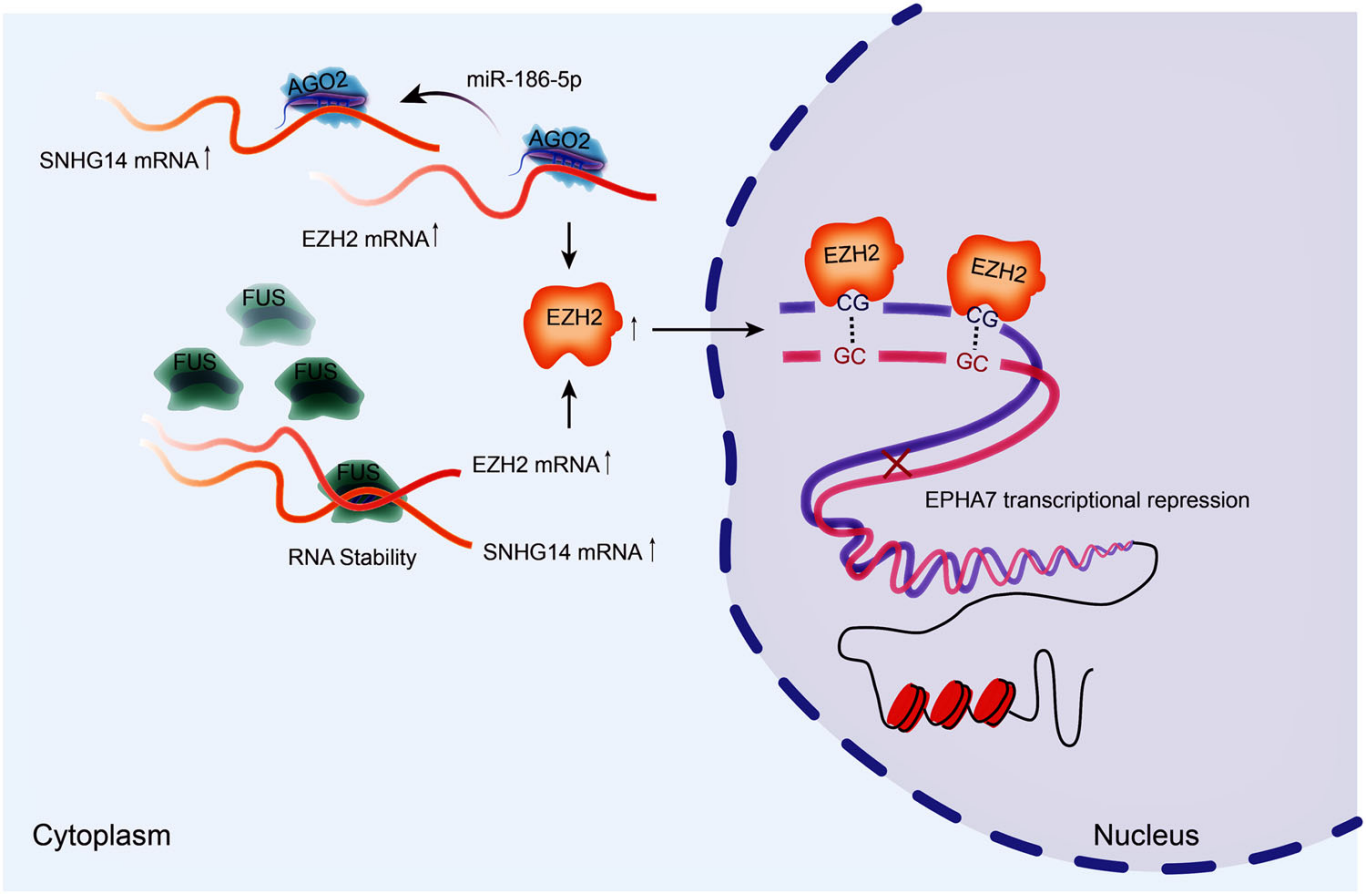
SNHG14 contributed to CRC progression through epigenetically silencing EPHA7 by upregulating EZH2 via recruiting FUS and sequestering miR-185-5p

On the whole, this study unmasked that SNHG14 aggravates CRC progression through inhibiting EPHA7 by upregulating EZH2 via recruiting FUS and absorbing miR-186-5p (Fig. 7), suggesting a new road for CRC diagnosis and treatment.
